# Artificial intelligence approaches in biological age prediction: current status and challenges

**DOI:** 10.3389/bjbs.2026.16141

**Published:** 2026-06-10

**Authors:** Guangjun Wang, Pengcheng Ding, Zihui Li, Qingfeng Tang, Liping Tu, Tao Jiang, Liangliang Zhang, Benyue Su, Jiatuo Xu, Hui An

**Affiliations:** 1 Digital and Intelligent Health Research Center, Anqing Normal University, Anqing, China; 2 School of Traditional Chinese Medicine, Shanghai University of Traditional Chinese Medicine, Shanghai, China; 3 School of Mathematics and Computer Science, Tongling University, Tongling, China; 4 Health Management and Physical Examination Center, Xiangyang Central Hospital, Affiliated Hospital of Hubei University of Arts and Science, Xiangyang, China

**Keywords:** age, aging, aging biomarker, biological age, chronological age, predication model

## Abstract

Biological age (BA) prediction has emerged as a critical research frontier for evaluating individual health status and aging trajectories beyond chronological age (CA). Recent advances in artificial intelligence (AI) have substantially accelerated this field by enabling the integration and interpretation of complex, multimodal biological data. This review provides a systematic overview of AI-driven approaches to BA prediction, covering key components including biomarker selection, feature engineering, model development, bias correction, and performance evaluation. We further highlight the growing recognition of asynchronous aging, a phenomenon in which different organs or physiological systems age at distinct rates, and discuss how AI—particularly deep learning and multimodal fusion—offers powerful tools for capturing such system-specific aging patterns. We summarize current methodologies ranging from traditional machine learning algorithms to advanced neural architectures capable of modeling nonlinear and heterogeneous aging processes. The expanding applications of AI-based BA models in disease risk assessment, geriatric evaluation, and population health monitoring are also examined. Despite rapid methodological progress, significant challenges persist, including data heterogeneity, limited model generalizability, insufficient interpretability, and barriers to clinical translation. Addressing these issues will require standardized methodological practices, robust validation across diverse populations, and the development of interpretable and equitable AI systems. Future research should prioritize the integration of multi-omics and longitudinal datasets with AI-driven analytical frameworks to establish reliable, system-level, and clinically actionable BA prediction models that account for asynchronous aging.

## Introduction

Aging is a universal yet highly heterogeneous biological process characterized by progressive functional decline and increased susceptibility to disease [1]. Chronological age (CA), defined simply as time elapsed since birth, fails to capture the substantial inter-individual variability in aging rates or the cumulative influence of genetic, environmental, and lifestyle factors on biological deterioration [2]. Individuals of identical CA often exhibit markedly different physiological states and disease risks, limiting the utility of CA for health assessment, risk stratification, and prognosis [3]. These limitations have motivated the search for alternative metrics that more accurately reflect the biological processes underlying aging.

Biological age (BA) emerged to address this gap as an objective, quantitative measure of physiological aging that integrates multidimensional biomarkers—including DNA methylation patterns, cellular senescence indicators, and organ-level functional parameters—into a unified framework [4]. Empirical evidence shows that individuals with the same CA may differ in BA by up to two decades, underscoring the inadequacy of CA as a surrogate for biological function [5]. Unlike CA, which is linear and irreversible, BA is dynamic and potentially modifiable, reflecting both current biological state and responsiveness to behavioral or therapeutic interventions. By capturing person-specific variation attributable to genetic predisposition (estimated to explain approximately 20– 30% of aging variability) and environmental exposures, BA correlates more strongly than CA with functional decline and mortality outcomes [6–10]. Since its conceptual articulation by Comfort in 1969, BA has reframed aging assessment from time elapsed to biological function, enabling more precise evaluation of health status and intervention efficacy [11–13].

Methodological advances have rapidly expanded BA estimation from single-domain biomarkers to integrative models informed by multi-omics and physiological data. DNA methylation–based clocks represent a major milestone in this evolution, demonstrating strong associations with cardiovascular risk, disease incidence, and long-term outcomes [14]. Longitudinal cohort studies further show that BA models outperform CA-based baselines in identifying individuals at elevated risk for age-related diseases [15]. At the same time, aging is increasingly recognized as an asynchronous process, in which different organs, tissues, and biological systems age at distinct rates within the same individual [16]. This intrinsic heterogeneity challenges traditional single-value BA estimates and necessitates modeling approaches capable of capturing nonlinear, system-specific aging dynamics. Artificial intelligence (AI) and machine learning techniques enable the integration of dozens to hundreds of biomarkers, offering powerful tools to model complex aging trajectories and improve prediction of healthspan and lifespan [17–19].

**FIGURE 1:**
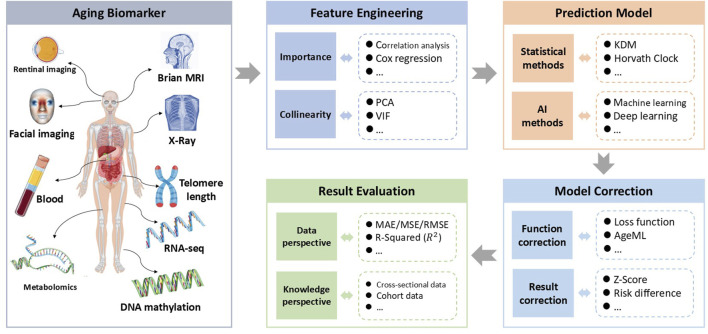
The general process of biological age prediction, mainly includes five parts: Aging biomarkers, feature engineering, prediction model, model correction, and result evaluation. Aging biomarkers: selecting different aging biomarkers according to different research purposes. Feature engineering: further processing the selected aging biomarkers. Prediction model: establishing a model to predict biological age based on the processed aging biomarkers. Model correction: correcting the bias of the prediction model. Result evaluation: evaluating the effectiveness of the prediction model.

In this review, we synthesize the methodological landscape of BA estimation with a particular focus on AI-driven approaches and their current limitations. As illustrated in Figure 1, we organize BA modeling into five interconnected components: (1) biomarker identification and validation, including emerging senescence-related markers; (2) feature representation and selection for high-dimensional omics data; (3) predictive modeling using regression, machine learning, and deep learning techniques; (4) model refinement to mitigate overfitting, bias, and population heterogeneity; and (5) evaluation and translation using standardized metrics for clinical applicability. By framing BA estimation within this structured pipeline, we highlight both the transformative potential of AI-based aging clocks and the key challenges that must be addressed to enable robust, interpretable, and clinically actionable BA assessment in precision medicine and public health [20].

## Aging biomarkers

Aging biomarkers provide the biological foundation for estimating BA beyond CA by quantifying molecular damage, systemic dysregulation, and organ-level structural or functional decline. In line with the heterogeneous and asynchronous nature of aging, contemporary BA research increasingly adopts a multimodal perspective, integrating biomarkers across molecular, physiological, and imaging scales. Rather than treating these markers in isolation, AI–based frameworks enable their joint modeling to capture nonlinear interactions and system-specific aging trajectories. Table 1 presents a tiered summary of representative aging biomarkers across these scales, highlighting their complementary roles in characterizing biological aging from molecular alterations to organ-level phenotypes.

**TABLE 1:** Representative aging biomarkers organized by biological tier for biological age prediction.

Biomarker	Key characteristics	Common AI models	References
Molecular-level biomarkers			
DNA methylation	High predictive accuracy; cross-tissue clocks (horvath, hannum, PhenoAge, GrimAge); limited interpretability	Elastic-net, ML, DL	[14, 21–23]
Telomere length	Replicative aging marker; associated with disease and mortality; lifestyle-sensitive	KDM, ML	[28, 29, 31, 62]
RNA-seq	Genome-wide expression profiling; captures pathway-level aging signals	ML, DL	[63, 64]
Metabolomics	Functional downstream readout; environmentally sensitive; standardization challenges	PCA, KDM, ML	[34, 65]
System-level biomarkers			
Blood tests	Multi-organ integration; inflammation, metabolic and hematologic indicators	KDM, MLR, DL	[13, 41]
Imaging-based biomarkers			
Brain MRI	Structural and functional brain aging; well-validated brain-age paradigm	CNN, transformer	[49, 51]
X-rays	Bone and dental structural aging; high availability	ML, CNN	[53, 54]
Facial imaging	Non-invasive; reflects lifestyle and cutaneous aging	CNN, transformer	[57, 58]
Retinal imaging	Microvascular and neural aging; scalable population screening	CNN, DNN	[60, 61]

At the molecular level, biomarkers reflect core cellular and biochemical processes underlying aging. DNA methylation (DNAm)–based epigenetic clocks, including Horvath’s, Hannum’s, PhenoAge, and GrimAge, represent the most accurate single-modality estimators of BA, showing robust associations with mortality, disease risk, and healthspan [14, 21–25]. However, their mechanistic interpretability remains limited, partly due to the integration of diverse environmental and genetic influences. For example, longevity-associated loci such as *APOE* modulate epigenetic age acceleration, with the ε 4 allele linked to faster biological aging and ε 2 showing relative protection [26, 27]. Telomere length reflects replicative senescence and mortality risk while exhibiting substantial inter-individual variability [28–31]. In parallel, conserved regulators such as *FOXO3* in the insulin/IGF-1 pathway influence oxidative stress resistance, inflammation, and telomere maintenance, thereby shaping molecular aging trajectories [32, 33]. Complementary transcriptomic and metabolomic signatures further capture age-related shifts in gene regulation and metabolism, and machine learning models trained on multi-omic data achieve BA prediction errors of approximately 4–5 years [34–36].

At the system level, blood-based biomarkers offer a clinically scalable and integrative view of organismal aging [37]. Inflammatory markers such as C-reactive protein and interleukins capture the phenomenon of inflammaging, while metabolic, hepatic, renal, and hematologic indices jointly reflect multi-organ functional decline [38–40]. Statistical approaches such as multiple linear regression and the Klemera–Doubal method (KDM), as well as deep learning–based “blood clocks,” transform multivariate laboratory panels into BA estimates with prediction errors typically within approximately 5 years [13, 41–43]. Importantly, composite system-level models acknowledge asynchronous aging across physiological systems, improving robustness and interpretability when compared with single-marker approaches [36, 44]. Large-scale electronic health record (EHR) have recently enabled the development of longitudinal, clinically deployable biological aging clocks that integrate routine healthcare data and predict adverse outcomes at population scale [37, 45].

In addition to molecular and system-level biomarkers, genetic background contributes substantially to inter-individual variability in aging trajectories, with heritability estimates for lifespan commonly reported at approximately 20%–30% in population-based studies [46]. Variants in longevity-associated genes such as *APOE* and *FOXO3* have been consistently linked to aging-related phenotypes. The *APOE* ε 4 allele confers increased risk of neurodegenerative disease and reduced survival, whereas *FOXO3* polymorphisms show reproducible associations with human longevity across diverse populations [32, 46]. Beyond inherited susceptibility, longitudinal clinical and biomarker trajectories are strongly associated with morbidity and mortality risk. Biological age acceleration measures and dynamic changes in physiological markers have been shown to predict healthspan and lifespan outcomes [5, 47]. However, most current AI-based BA models rely primarily on biomarker patterns aligned with chronological age and rarely incorporate genotype information or structured longitudinal clinical context into model architectures. Integrating polygenic risk scores (PRS), gene–environment interactions, and harmonized longitudinal clinical data into BA frameworks may improve personalization and help distinguish intrinsic biological aging from disease-driven acceleration [36].

Imaging biomarkers provide a complementary organ-specific perspective on aging by quantifying structural and functional changes at the tissue level [48]. Brain MRI–based aging models capture age-related alterations in cortical thickness, gray and white matter integrity, and network organization, with deep learning architectures achieving mean absolute errors as low as 2–3 years and showing strong associations with cognitive decline and neurodegenerative risk [49–52]. Other imaging modalities extend BA estimation beyond the brain: dental and skeletal X-rays quantify mineralized tissue aging [53–55], facial imaging captures cutaneous and craniofacial aging influenced by lifestyle and environmental exposure [56–58], and retinal imaging provides a non-invasive window into microvascular and neural aging [59–61]. These modalities are particularly well suited for AI-based analysis due to their high dimensionality and scalability.

Collectively, aging biomarkers span a continuum from molecular damage and genetic susceptibility to system-wide physiological dysregulation and organ-level structural decline. Although epigenetic clocks and brain-age models provide high predictive accuracy within individual modalities, aging is inherently multidimensional and asynchronous. AI-driven multimodal fusion that integrates molecular, imaging, genetic, and longitudinal clinical features may therefore yield more comprehensive and clinically meaningful BA estimates. Continued progress will require standardized data processing pipelines, rigorous longitudinal validation, improved model interpretability, and careful handling of genetic and clinical heterogeneity. With these advances, BA may transition from a descriptive aging indicator to a clinically actionable metric supporting personalized healthy aging strategies.

## Feature engineering

Feature engineering is central to BA modeling because it determines how heterogeneous aging biomarkers are converted into representations that are robust, informative, and suitable for downstream learning. Unlike conventional risk modeling, BA estimation must integrate signals across molecular mechanisms, systemic physiology, and organ-level phenotypes; moreover, aging is increasingly recognized as an *asynchronous* process, in which organs and biological systems may age at distinct rates within the same individual. This intrinsic heterogeneity makes feature engineering not merely a preprocessing step, but a key design decision that shapes whether BA models can capture system-specific aging trajectories while remaining generalizable and clinically interpretable [13, 22, 66].

In practice, feature engineering for BA prediction can be viewed as a pipeline consisting of (1) modality-aware preprocessing and representation construction, (2) feature screening and dimensionality reduction for stability and interpretability, and (3) multimodal fusion to support holistic and system-level BA estimation. At the molecular level, biomarkers such as DNA methylation loci, transcriptomic profiles, metabolomic signatures, and telomere length encode fundamental cellular aging mechanisms but are typically high-dimensional and noisy. These data commonly require normalization (e.g., z-score or quantile normalization) and batch-effect mitigation to ensure comparability across samples and platforms [27, 36]. Dimensionality reduction techniques such as principal component analysis (PCA) and, increasingly, autoencoder-based representation learning are then used to extract compact latent factors that summarize dominant age-related variation while suppressing technical noise [37, 66–69]. At the system level, blood- and physiology-derived indicators offer scalable clinical features that reflect multi-organ regulatory decline; however, they often exhibit redundancy and confounding, making feature selection essential for interpretability and transportability [42, 43, 70]. For imaging modalities (e.g., brain MRI, retinal images, facial photographs), deep neural networks provide end-to-end feature extraction by encoding complex morphological patterns into low-dimensional embeddings that can be aligned with aging outcomes [51, 52, 58, 61, 71, 72].

Classical statistical screening methods remain widely used because they provide transparent and clinically intuitive feature panels. Correlation with chronological age (CWCA) is often adopted when BA ground truth is unavailable, using CA as a proxy to filter biomarkers with monotonic age trends [13, 73, 74]. Cox proportional hazards regression further prioritizes biomarkers most predictive of survival or health outcomes, thereby linking BA construction to clinically meaningful endpoints [5, 75]. Multicollinearity diagnostics such as the variance inflation factor (VIF) reduce redundancy and improve interpretability, and have been used extensively in NHANES-style BA modeling to refine biomarker sets [22, 76–78]. PCA offers a complementary strategy by projecting correlated biomarkers into orthogonal components and extracting latent aging factors, which can improve stability in high-dimensional settings and facilitate model transfer [66, 67, 79, 80]. These methods collectively establish a baseline feature engineering toolkit for BA modeling; however, their reliance on linear assumptions may limit the capture of nonlinear and hierarchical interactions among biomarkers.

Recent advances in artificial intelligence have shifted feature engineering from manual screening toward *representation learning* and *multimodal fusion*. Autoencoders and variational autoencoders (VAEs) learn compact and noise-robust embeddings from omics or imaging data, preserving nonlinear structure that conventional reductions may overlook [69, 81]. Graph neural networks provide an additional mechanism for modeling biomarker dependencies and biological structure, such as gene co-expression networks or pathway connectivity, enabling relational reasoning beyond independent-feature assumptions [82]. Attention-based models and Transformers further support cross-feature interaction modeling and facilitate interpretability by learning feature importance weights dynamically, making them increasingly relevant for heterogeneous biomarker integration [51, 83]. Importantly, these AI approaches are well suited to the challenge of asynchronous aging: they can encode organ- or system-specific representations and then fuse them into a unified BA estimate, allowing the model to reflect differential aging rates across biological subsystems [52, 58, 61].

Despite their promise, AI-based feature engineering introduces several unresolved challenges that directly affect model validity and translation. First, multimodal datasets remain limited and heterogeneous, and representation learning models can be sensitive to batch effects, missing modalities, and population shifts, reducing generalizability across cohorts [27, 36]. Second, feature embeddings learned by deep models may lack biological interpretability, complicating mechanistic understanding and clinical trust [5, 13]. Third, bias can be introduced when feature construction pipelines are optimized for prediction accuracy but fail to account for confounding structure (e.g., sex, ancestry, socioeconomic factors), leading to systematic error in BA estimates. Addressing these challenges will require standardized preprocessing protocols, robust validation across diverse populations, and the development of interpretable and bias-aware feature learning strategies that can support equitable, clinically actionable BA prediction.

Genetic variants and structured clinical history require specialized representation strategies within AI-based BA pipelines. PRS provide a compact representation of inherited susceptibility to age-related diseases and have been increasingly used to stratify aging risk and healthspan outcomes [84]. Meanwhile, longitudinal EHR-derived features can encode cumulative multimorbidity and treatment exposure, offering dynamic context for BA modeling [5, 85]. Advanced AI architectures—including graph neural networks and multimodal transformer models—are particularly suited to modeling gene–environment interactions and nonlinear dependencies between inherited risk and physiological aging signals [82, 86]. Systematically integrating genetic susceptibility and clinical trajectories into BA models may improve robustness and enable clearer differentiation between intrinsic aging processes and pathology-related acceleration.

## Prediction models

A broad range of models have been developed for BA prediction, progressing from regression-based statistical frameworks to modern deep learning architectures. In practice, model selection is primarily determined by data modality, feature dimensionality, and the trade-off between interpretability and predictive performance. To better reflect this evolution, we categorize BA prediction models into two major groups: machine learning approaches (including traditional regression-based methods) and deep learning approaches.

### Machine learning approaches

Machine learning (ML) provides a flexible data-driven framework for BA prediction and encompasses both classical statistical methods and modern nonlinear algorithms. Early BA estimators are often derived from multivariate regression frameworks that combine clinical biomarkers into a single composite index. A representative example is the KDM, which estimates BA through a weighted multivariate regression scheme, assigning each biomarker a weight inversely proportional to its measurement error [42, 87]. KDM-based BA acceleration has been shown to associate with morbidity, mortality, and lifestyle-related risk factors in large-scale cohorts [88, 89]. Despite its interpretability, such regression-based models are constrained by linear assumptions and limited adaptability to heterogeneous or high-dimensional data.

Modern ML models extend beyond linear constraints by capturing nonlinear relationships among complex biomarker sets. Algorithms such as elastic net regression, random forests, and support vector machines (SVMs) have been widely applied to high-dimensional omics, proteomic, and imaging datasets [90]. For instance, Ganaie et al. employed an improved least-squares twin support vector regression to estimate brain age from MRI data, achieving a mean absolute error (MAE) of approximately 3 years [72]. Similarly, proteomic clocks trained using ML models have demonstrated strong predictive accuracy and revealed systemic aging patterns across tissues and organ systems [91]. Overall, ML approaches offer improved flexibility and scalability relative to traditional regression methods, but they often rely on handcrafted features, careful hyperparameter tuning, and may have limited interpretability in high-dimensional settings.

### Deep learning and emerging trends

Deep learning (DL) has increasingly become central to BA prediction due to its capability for hierarchical representation learning and end-to-end feature extraction. Neural architectures including convolutional neural networks (CNNs), recurrent neural networks (RNNs), and variational autoencoders (VAEs) have demonstrated strong performance in modeling nonlinear aging patterns in omics, imaging, and physiological signals [92, 93]. Unlike conventional ML pipelines, DL models can automatically learn latent aging representations without manual feature engineering. For example, Galkin et al. developed DeepMAge, a neural network-based epigenetic clock trained on over 4,000 blood methylomes, achieving an MAE of 2.77 years and outperforming regression-based baselines [69]. In neuroimaging, He et al. proposed a global–local transformer framework integrating global contextual information with local fine-grained features, reducing MAE to 2.7 years in brain age estimation [51].

Recent methodological advances highlight a shift toward multimodal and self-supervised paradigms. Transformer-based models are increasingly adopted for capturing long-range dependencies and cross-modal interactions, enabling unified latent representations across diverse biological inputs. Multimodal transformer architectures integrating methylation, transcriptomic, and imaging signals have shown improved prediction accuracy and interpretability [86, 94, 95]. Meanwhile, contrastive learning and other self-supervised objectives have emerged as powerful strategies to disentangle aging signals from confounding noise and enhance robustness across datasets [96, 97]. Additionally, multitask learning and adversarial autoencoder frameworks have been proposed to jointly predict BA and auxiliary outcomes such as sex, disease risk, or mortality, improving regularization and domain transferability [98].

Overall, BA prediction has transitioned from biomarker-based regression estimators toward scalable, multimodal deep architectures that learn biologically grounded aging representations. Future research should emphasize multimodal integration, interpretability, and validation across diverse populations to ensure translational reliability of biological aging models.

## Model correction

In statistical and machine learning–based BA estimation, model correction serves as a crucial post-prediction process that improves accuracy, reduces systematic bias, and enhances generalizability across heterogeneous populations. Biological and environmental variability—such as genetic background, ethnicity, and lifestyle—can introduce consistent deviations between predicted BA and CA. Without correction, these biases may propagate into downstream analysis, leading to inaccurate or inequitable outcomes across demographic subgroups [99]. For example, race- and sex-specific biomarker distributions may alter regression slopes or shift prediction intercepts, motivating population-aware calibration. Correction is therefore essential for ensuring fairness, interpretability, and translational reliability of BA estimation in real-world settings.

A second motivation relates to dataset transferability. Models trained on a specific cohort often generalize poorly to external datasets due to overfitting to cohort-specific noise, measurement protocols, or confounding variables [100]. Correction frameworks mitigate this issue by recalibrating model outputs to new distributions while preserving biologically meaningful deviations. Importantly, even when the overall predictive error is low, systematic deviations may persist—most notably overestimation in younger individuals and underestimation in older ones, often referred to as the age–delta correlation (ADC) or regression-to-the-mean problem [101]. Model correction, applied either during training or *post hoc*, aims to align predicted and true age distributions and remove these structural biases. Representative correction strategies are summarized in Table 2.

**TABLE 2:** Representative model correction methods for biological age estimation.

Method	Conceptual basis	References
Skewed loss function	Replaces symmetric regression losses with asymmetric, age-dependent penalties to eliminate bias during training	[101–103]
AgeML	Applies linear recalibration of predicted vs. true age to remove regression-to-the-mean effects	[104–106]
Z-score correction	Standardizes biomarkers or predicted ages to population-level mean and variance, minimizing demographic bias	[7, 81, 107]
Risk-difference	Uses reference risk functions or expected age indices (e.g., vascular age) for relative BA estimation	[107–109]

### Loss function–based correction

Loss function modification provides an intrinsic correction mechanism during model training. Conventional regression objectives such as mean absolute error or mean squared error treat under- and overestimation symmetrically, which can reinforce age-dependent biases in BA prediction. To address this, recent approaches introduce skewed or asymmetric losses that apply age-dependent weighting to penalize systematic underestimation in older individuals and overestimation in younger ones [101, 103].

In the AccelerAge framework, Wang et al. proposed an adaptive penalty strategy in which the magnitude of the gradient is adjusted according to both the subject’s age and the direction of the prediction error. This design explicitly targets the ADC phenomenon by discouraging structured error patterns and driving the correlation between prediction residuals and CA toward zero. Evaluations on neuroimaging datasets such as Cam-CAN and ABIDE demonstrated that asymmetric-loss training substantially reduced systematic bias while maintaining competitive accuracy. Similar bias-aware loss formulations have also been applied in MRI-based BA modeling [102], improving stability at the extremes of the age spectrum and supporting fairer performance across age groups.

### AgeML linear recalibration

AgeML provides an interpretable and statistically grounded post-processing correction. It assumes that predicted age and true CA exhibit an approximately linear relationship and performs a regression-based recalibration to remove regression-to-the-mean bias frequently observed in uncorrected BA estimates [104, 105]. In practice, AgeML fits a linear model relating predicted age to CA on the reference dataset and then uses the fitted slope and intercept to adjust the predicted values. After correction, the adjusted predictions are designed to be unbiased with respect to CA, while the remaining residual reflects age acceleration or deceleration relative to normative expectations.

This approach is attractive because it is transparent, computationally inexpensive, and applicable across different BA modalities. Condado et al. showed that AgeML can remove bias consistently across sex and disease stratified cohorts when a healthy reference population is available [105]. As a result, linear recalibration has become a widely used component in clinical BA pipelines, particularly for epigenetic and imaging-based clocks. A recent extension of this approach was applied in an ECG-based age prediction study, where linear recalibration was first used to remove the global age-related bias, followed by an age-stratified mean-centering step to eliminate residual nonlinear associations with CA, achieving a fully age-independent deviation metric $\left(PAD_{bc}\right)$ [106].

### Z-score correction

Z-score correction is among the simplest and most generalizable calibration techniques. It rescales biomarkers or predicted BA values by centering them to a reference mean and scaling them by the corresponding standard deviation, yielding standardized outputs that are comparable across cohorts. In BA modeling, z-score–based correction is commonly applied to reduce demographic and cohort-dependent bias by normalizing interindividual variability and harmonizing distributions across populations [81].

Peters et al. [7] and Oh et al. [107] demonstrated that such normalization improves robustness and cross-population transferability, particularly for biomarkers with high variance and strong population stratification. Recent work has further extended z-score calibration to multi-organ aging frameworks, enabling standardized comparison of organ-specific BA metrics across demographic groups and supporting downstream risk stratification [107].

### Risk-difference

Beyond purely statistical normalization, risk-based calibration methods define BA in clinically interpretable terms by referencing expected age-dependent physiological profiles [110]. Tang et al. proposed vascular aging frameworks in which predicted physiological parameters are compared to their expected reference values at a given CA [108, 109]. The deviation between observed and expected values is then interpreted as accelerated or decelerated vascular aging and used to construct a corrected vascular age metric.

Such formulations enable risk-normalized calibration by explicitly mapping deviations in physiological function to interpretable BA adjustments, thereby aligning BA prediction with clinically meaningful outcomes [111]. Recent extensions have generalized this idea to organ-specific aging indices, including hepatic and renal aging, yielding unified risk-referenced frameworks that support precision medicine applications and cross-organ comparisons [107].

## Result evaluation

Rigorous evaluation of BA prediction models is essential for assessing their reliability, interpretability, and translational value. Unlike conventional regression tasks, BA estimation lacks a definitive biological ground truth, making evaluation inherently multidimensional. High numerical accuracy alone does not guarantee that a model captures meaningful aging processes or generalizes across populations. Accordingly, BA evaluation is commonly conducted from two complementary perspectives: a data-oriented statistical perspective and a knowledge-oriented biological perspective, as summarized in Table 3.

**TABLE 3:** Common biological age validity indicators.

Perspective	Evaluation indicator	Purpose	References
Data perspective	MAE, MSE, RMSE	Quantify prediction error relative to CA	[51, 52, 112, 113]
​	$R^{2}$	Assess variance explained and model fit	[7, 13, 114, 115]
Knowledge perspective	Cross-sectional analysis	Identify health differences across individuals or subgroups	[44, 47, 116, 117]
​	Longitudinal cohort analysis	Track individual aging trajectories and outcomes over time	[118–120]

### Data perspective

From the data-oriented perspective, BA prediction is typically evaluated using statistical regression metrics that quantify the deviation between predicted BA and CA, which serves as a proxy reference in the absence of a biological ground truth. Commonly used indicators include MAE, mean squared error (MSE), root mean squared error (RMSE), and the coefficient of determination $\left(R^{2}\right)$ [45, 121]. These metrics provide standardized and easily comparable measures of predictive performance across models and datasets.

Among these indicators, MAE is particularly favored for its interpretability and robustness to outliers, reflecting the average deviation of predictions in age-equivalent units. RMSE and MSE emphasize larger errors and are useful for identifying models prone to extreme misestimation, which is especially relevant in clinical risk stratification. Deep learning–based BA models, particularly in neuroimaging and multimodal settings, now routinely achieve RMSE values below 3–4 years, indicating substantial progress in predictive accuracy [51, 115].

The $R^{2}$ complements error-based metrics by quantifying how much inter-individual variation in aging is captured by the model. Higher $R^{2}$ values suggest stronger consistency between predicted and observed age distributions and better modeling of population-level aging variability. However, high statistical performance does not necessarily imply biological validity, motivating the need for complementary evaluation frameworks.

### Knowledge perspective

The knowledge-oriented perspective evaluates whether BA estimates reflect biologically meaningful aging processes rather than merely fitting chronological age. This perspective emphasizes interpretability, physiological relevance, and consistency with known aging patterns, and is commonly assessed through cross-sectional and longitudinal analyses.

Cross-sectional evaluation examines BA differences across individuals or subgroups at a single time point. A widely used indicator is the BA delta, defined as the deviation between predicted BA and CA. Positive deviations are interpreted as accelerated aging, while negative deviations indicate relative biological youth. Cross-sectional analyses have been used to identify health disparities associated with disease status, frailty, lifestyle factors, and socioeconomic conditions [44, 117]. Despite their scalability and practicality, cross-sectional studies rely on the assumption that associations between biomarkers and CA reflect true aging processes, an assumption that may be violated by cohort effects or confounding factors [113].

Longitudinal cohort studies provide a stronger biological validation framework by tracking intra-individual BA trajectories over time [122]. Fixed cohorts, in particular, enable direct assessment of whether BA acceleration predicts future health decline, disease onset, or mortality. Numerous studies have demonstrated that longitudinal BA changes are associated with cognitive impairment, multimorbidity, and survival outcomes, supporting BA as a prognostic aging marker [118, 120]. For example, cohort analyses of centenarian populations have revealed stable molecular and physiological signatures associated with exceptional longevity, confirming that longitudinal evaluation captures genuine biological aging trajectories [119]. EHR further extend longitudinal validation by providing real-world clinical evidence [123]. Large-scale EHR data allow assessment of whether BA acceleration predicts incident disease, multimorbidity, and mortality across heterogeneous populations [37]. Demonstrated associations between elevated BA and adverse outcomes support its prognostic and translational relevance beyond CA. Despite challenges such as missing data and coding bias, EHR-based analyses strengthen the link between biological aging signals and tangible health consequences [85].

Taken together, data-oriented metrics provide standardized benchmarks for model comparison, while knowledge-oriented evaluation establishes biological plausibility and translational relevance. Robust BA assessment therefore requires the integration of statistical accuracy and biologically informed validation, further strengthened by real-world clinical evidence derived from longitudinal cohorts and EHR-based outcome analyses.

## Challenges

BA is an indicator that estimates the degree of physiological aging by assessing an individual’s biological state. Compared with CA, BA more accurately reflects health status and aging rate, and therefore plays a crucial role in aging research and clinical practice. With the rapid development of AI and high-throughput biomedical technologies, BA prediction has gradually shifted from traditional statistical models to data-driven AI-based approaches. These advances have significantly improved predictive performance and enabled the integration of multi-modal data. However, the increasing complexity of AI-based BA models has also introduced new methodological and conceptual challenges. In particular, issues related to biomarker selection, model interpretability, validation strategies, generalizability, and the characterization of asynchronous aging remain unresolved. This section discusses the key challenges of BA research from five interconnected perspectives, providing a reference for future in-depth studies.

### How to select representative aging markers under multi-modal and asynchronous aging frameworks

Recent AI-based BA studies leverage a wide range of biomarkers, including blood tests, DNA methylation, transcriptomics, metabolomics, medical imaging (e.g., brain MRI, X-rays), facial and retinal images, and wearable sensor data [124]. Deep learning models are particularly effective at extracting latent features from high-dimensional data, enabling multi-modal fusion for BA prediction. However, the impact of biomarker selection and combination on BA estimation remains unclear [125].

Moreover, aging is increasingly recognized as an asynchronous and organ-specific process, in which different tissues and systems age at distinct rates within the same individual [107]. Current AI models often aggregate heterogeneous biomarkers into a single BA output, potentially obscuring organ-level aging patterns and biological heterogeneity. In addition, substantial variability in biomarker panels across studies limits reproducibility and cross-cohort comparability. Therefore, a key challenge lies in developing AI-driven biomarker selection strategies that are not only predictive but also biologically interpretable, capable of capturing asynchronous aging processes across organs and systems.

### How to analyze the logical structure of BA to improve the interpretability of AI-based models

Existing BA prediction methods range from traditional models such as the KDM and epigenetic clocks (e.g., Horvath Clock) to machine learning and deep learning approaches. While AI models excel at handling complex, nonlinear relationships in large-scale datasets, they often function as “black boxes,” lacking explicit representation of the biological logic underlying BA [126]. This opacity limits their acceptance in clinical settings, where interpretability and causal insight are critical.

Additionally, the absence of a universally accepted gold standard for BA poses a fundamental challenge. Most supervised AI models use CA as the training label, implicitly assuming that deviations from CA reflect biological aging. This assumption may bias models toward chronological patterns rather than true aging mechanisms and increases the risk of overfitting [101]. Consequently, improving BA prediction requires not only algorithmic innovation but also a clearer conceptual framework that links AI-derived features to biological aging pathways and asynchronous aging structures.

### How to scientifically validate AI-based BA models beyond chronological age prediction

BA remains a latent construct without a direct measurement standard. Although CA is commonly used as a proxy for model evaluation, this approach fails to capture health-related deviations that define biological aging. Different AI models may yield substantially different BA estimates for the same individual, complicating model comparison and selection [127].

Current evaluation metrics such as MSE, RMSE, MAE, and $R^{2}$ primarily assess numerical agreement with CA and do not adequately reflect biomedical relevance. Longitudinal validation using morbidity, mortality, or functional decline as endpoints is more informative but requires extensive time and resources. Cross-sectional studies, which dominate current AI-based BA research, cannot fully capture temporal aging trajectories or asynchronous aging dynamics [108]. Therefore, establishing a systematic and multi-level validation framework that integrates clinical outcomes, longitudinal changes, and organ-specific aging indicators remains a major challenge.

### How to enhance the accuracy, robustness, and generalizability of AI-driven BA models

To facilitate the clinical translation of AI-based BA models, future research must move beyond incremental performance gains and address foundational challenges in model design, data integration, and validation. Incorporating established aging mechanisms—such as inflammation, metabolic dysregulation, cellular senescence, and epigenetic drift—can improve biological interpretability and reduce spurious associations [45]. Aging should also be modeled as a multidimensional and asynchronous process, with multi-omics and multi-modal data integration enabling the capture of organ- and system-specific aging trajectories [16]. Interpretable and explainable AI approaches are essential for clinical trust and adoption, while robust generalization requires large-scale, longitudinal, and demographically diverse cohorts [128]. Finally, standardized protocols for data processing, model training, and evaluation are critical for reproducibility and cross-study comparability.

By systematically addressing these challenges, AI-based BA models can move beyond mere CA prediction toward a more biologically grounded and clinically actionable understanding of human aging.

### Integration of genetic susceptibility and medical history

Although genetic variation is estimated to explain a substantial proportion of aging heterogeneity, most AI-driven BA models do not explicitly model genotype or inherited susceptibility. Instead, models trained against chronological age risk conflating disease burden with intrinsic aging processes. Recent reviews emphasize that biological aging should be conceptualized as a multidimensional process shaped by genetic background, environmental exposure, and stochastic factors [16, 127].

Incorporating genotype information (e.g., *APOE* status, *FOXO3* variants, polygenic risk scores) alongside longitudinal medical history requires harmonized genomic and clinical datasets, careful control for population stratification, and bias-aware modeling strategies [36]. Developing unified frameworks that jointly model multimodal biomarkers, genetic predisposition, and clinical trajectories remains a major frontier in precision aging research.
